# EtSERPIN1 binding with chicken ANXA2 is essential for *Eimeria tenella* attachment and invasion process

**DOI:** 10.1186/s13567-025-01532-w

**Published:** 2025-07-10

**Authors:** Zengbao Wang, Taifeng Li, Yingying Jiang, Xue Wang, Hongmei Li, Xiaomin Zhao, Xiao Zhang, Ningning Zhao

**Affiliations:** 1 Department of Preventive Veterinary Medicine, College of Veterinary Medicine, Shandong Agricultural University, 7 Panhe Street, Tai’an, 271017 Shandong China; 2 Shandong Provincial Key Laboratory of Zoonoses, Shandong Agricultural University, Tai’an, Shandong China; 3 The Affiliated Taian City Centeral Hospital of Qingdao University, Tai’an, Shandong China

**Keywords:** *Eimeria tenella*, EtSERPIN1, GgANXA2, adhesion, invasion

## Abstract

Serpin protease inhibitors (SERPIN) in protozoa play crucial roles in various biological processes, including the invasion of host cells. However, the precise roles and molecular mechanisms underlying SERPIN-mediated invasion of parasite remain poorly understand. In this study, we provide evidence that surface-expressed *Eimeria tenella* SERPIN1 (EtSERPIN1) on sporozoites is involved in adhesion and invasion processes. To elucidate the molecular target responsible for mediating EtSERPIN1-induced invasion, we utilized GST pull-down and yeast two-hybrid assays to identify host cell membrane proteins interacting with EtSERPIN1. Our findings revealed an interaction between EtSERPIN1 and a membrane protein called annexin A2 (ANXA2). Furthermore, recombinant GgANXA2 was shown to bind to the sporozoite surface. Pre-treatment with anti-GgANXA2-specific antibody or recombinant GgANXA2 protein significantly inhibited EtSERPIN1 binding to host cells. Pre-incubation with recombinant GgANXA2 also reduced sporozoite infection in both DF-1 cells and chickens. These results suggest that the interaction between EtSERPIN1 and GgANXA2 plays a critical role in both adhesion and invasion processes of *E. tenella* sporozoites. Finally, we investigated the impact of recombinant GgANXA2 and EtSERPIN1 proteins on *E. tenella* infection. Our results demonstrate that incubation with GgANXA2 protein significantly attenuated sporozoite infectivity, as evidenced by a significant reduction in parasite burden within the chicken cecum. Immunization with recombinant EtSERPIN1 exhibited potent anti-*E. tenella* activity, with higher body weight gains, lower cecal lesions and oocyst output, as well as elevated levels of cecal mucosa antibodies. These findings suggest that targeting GgANXA2 through EtSERPIN1 mediates adhesion and invasion processes of *E. tenella*, highlighting its potential as a novel therapeutic target.

## Introduction

Avian coccidiosis, caused by intestinal infection with one or multiple *Eimeria* species, leads to great economic losses worldwide [1]. The current approach to controlling avian coccidiosis primarily relies on the utilization of anti-coccidial drugs and live coccidia vaccines. However, the use of anticoccidial drugs is increasingly limited due to issues such as drug resistance, residues, regulatory restrictions and limitations during animal feeding [2, 3]. Moreover, the elevated production cost and potential pathogenicity of live *Eimeria* strain vaccination have hindered their widespread application in the poultry industry [4]. A comprehensive understanding of coccidia-host interactions and molecular mechanisms will facilitate the development of novel control strategies.

The initial step in establishing an infection by *Eimeria* spp. involves the invasion of host cells, which consists of four sequential processes: attachment, apical reorientation, formation of a moving junction, and establishment of a protective parasitophorous vacuole (PV). These invasion steps are facilitated by proteins secreted from organelles located at the apical end of the parasite [5]. It is known that microneme proteins (MIC) have been identified as crucial players in parasite attachment progress [6, 7], relying on an assortment of domains such as apple, microneme adhesive repeat regions (MARR), integrin-like A, lectin and epidermal growth factor (EGF)-like domains [8–11]. However, knockout experiments targeting MIC only partially inhibit sporozoite invasion, indicating that other proteins may also be involved in parasite adhesion to host cells [12, 13]. One group of these proteins is serpin protease inhibitors (SERPIN), which have been reported to participate in the process of parasite adhesion and invasion [14, 15].

SERPIN are members of a highly conserved superfamily of proteins that exhibit a well-preserved tertiary structure and have functional presence across various organisms, ranging from viruses to mammals [16]. They function as potent inhibitors of serine proteases and play crucial roles in numerous fundamental biological processes, including blood coagulation, fibrinolysis, angiogenesis, programmed cell death, development, and inflammation [17]. *Toxoplasma gondii* SERPIN1 (TgPI) was initially identified in protozoan parasites [15, 18, 19]. Subsequently, several other SERPIN were discovered in *Neospora caninum*, *Entamoeba histolytica*, and *Eimeria* spp. [14, 15, 18, 20]. In *Eimeria* spp., the presence of SERPIN has been confirmed in both *E. tenella* and *Eimeria acervulina* through studies conducted by Jiang et al. [14] and Fetterer et al. [15]. Since then, the involvement of SERPIN in *Eimeria* spp. cell invasion has been established. However, the precise role played by SERPIN during the sporozoite invasion process remains unclear for *Eimeria* spp. Therefore, this study aims to identify membrane proteins interacting with *E. tenella* SERPIN1 (EtSERPIN1) using pull-down assays coupled with mass spectrometry analysis to elucidate the molecular mechanism underlying sporozoite invasion mediated by EtSERPIN1.

## Materials and methods

### Plasmids, yeast, parasites, cells and animals

The pCTCON2 and *Saccharomyces cerevisiae* EYB100 stored in our laboratory were used for the yeast surface display system and adhesion assay [11]. The wild type strain *E. tenella* Shandong strain-01 (SD-01) was isolated and stored in our laboratory [21]. Chicken embryo fibroblast cell line (DF-1) cells stored in our laboratory were maintained in Dulbecco minimal essential medium (DMEM) (Gibco, New York, NY, USA) containing 10% fetal bovine serum (FBS) (Biological Industries, Israel) and 1% penicillin–streptomycin (Gibco) at 37 °C in 5% CO_2_. All cells were confirmed to be negative for mycoplasma contamination using a mycoplasma detection kit (Cat# CA1080, Solarbio, Beijing, China).

BALB/c mice purchased from Jinan Pengyue Experimental Animal Breeding Co., Ltd were utilized for the production of polyclonal antibodies (pAb) as previously described [21]. Coccidia-free 1-day old Hy-Line layer chickens were purchased from the Dongyue Breeder Company (Tai’an, China). All animals were maintained under sterile conditions and provided with appropriate feed and water without the use of coccidiostats.

### Subcellular localization of EtSERPIN1

Indirect immunofluorescence assay (IFA) was performed according to the previously published protocol to determine the subcellular localization of EtSERPIN1 in *E. tenella* [11]. Briefly, *E. tenella* sporozoites, second-generation schizonts and gametophytes were fixed, permeabilized, and sequentially incubated with anti-EtSERPIN1 pAb and fluorescein isothiocyanate (FITC)-conjugated goat anti-mouse IgG antibody (Solarbio). Nuclei were stained with 4',6-diamidino-2-phenylindole (DAPI) (Solarbio). Images were visualized and acquired using fluorescence microscopy (Nikon-ECLIPSE; Japan).

### Sporozoite secretion assay

Sporulated oocysts were sterilized by treatment with 20% sodium hypochlorite for 10 min at room temperature. The oocysts were then mechanically disrupted by grinding with glass beads to release the sporocysts. Excystation of the sporozoites was achieved by incubating the sporocysts in sterile PBS containing 0.25% trypsin (Solarbio) and 0.25% sodium taurocholate at 41 °C for 40 to 60 min. The released sporozoites were collected by filtration through a G3 funnel. To activate the sporozoites, they were incubated with 400 nM of calcium ionophore A23187 (Aladdin, Shanghai, China) at 37 °C for 20 min. Following incubation, the supernatant was collected by centrifugation at 1000 rpm and used for the analysis of secreted proteins.

### Glutathione S-transferase (GST) pull-down assay

Preparation of membrane proteins from the chicken caecum epithelium: The cecal mucosa of healthy chickens was scraped and stored at -80 °C. Membrane proteins from the chicken caecum epithelium were extracted using the Membrane Protein Extraction kit (Solarbio), according to the manufacturer’s instructions.

The GST pull-down assay was performed according to a previously described method [22]. Firstly, recombinant GST-tag EtSERPIN1 protein (rGST-EtSERPIN1) was expressed in BL21. Then, glutathione resins (Solarbio) were separately incubated with rGST-EtSERPIN1 or GST-tag (control) at 4 °C for 5 h. After three washes to remove non-resin bound proteins, the glutathione resins were mixed with membrane protein from the chicken caecum epithelium at 4 °C for 2 h, followed by elution of the bait-prey mixture using elution buffer (10 mmol/L reduced glutathione, pH 8.0). Finally, the samples were separated by 12% sodium dodecyl sulfate–polyacrylamide gel electrophoresis (SDS-PAGE).

### Mass spectrometry

The silver staining and mass spectrometry were performed according to previously described methods [23]. Briefly, the interaction complexes of rGST-EtSERPIN1 group and GST-tag group were separated by SDS-PAGE respectively and subjected to Fast stain silver kit according to the manufacturer’s instructions (Beyotime, Shanghai, China). Subsequently, the interaction protein bands of rGST-EtSERPIN1 were excised from the gel. Then the samples were trypsin-digested, and analyzed using mass spectrometry (MS) based on a standardized protocol employing QSTAR XL instrument (Applied Biosystems, CA, USA). All experimental procedures were performed by the BPI Genomics Company (Shenzhen, China).

### Yeast two-hybrid verification test (Y2H)

The yeast two-hybrid verification assay was carried out according to previously described methods [22]. Briefly, the coding sequence (CDS) of EtSERPIN1 was cloned into the pGBKT7 plasmid to construct the bait plasmid (pGBKT7-EtSERPIN1). The CDS sequence of chicken annexin A (*ANXA*) genes, including GgANXA 2, 3, 5, and 13, were cloned into pGADT7 plasmid as potential prey. Subsequently, co-transformation of pGBKT7-EtSERPIN1 with either pGADT7-GgANXA2 or pGADT7-GgANXA3 or pGADT7-GgANXA5 or pGADT7-GgANXA13 into Y2H Gold strain was carried out using the Y2HGold-GAL4 Y2H interaction proving kit (Coolaber, Beijing, China). The interaction between the EtSERPIN1 and potential prey proteins was confirmed by the growth of co-transformants on DDO/X/A and QDO/X/A plate at 30 °C for 3-5 days. The positive control used was pGBKT7-p53.

### Western blot

The western blot assay was conducted as previously described [24]. The isolated protein or GST pull-down samples were resolved on a 12% SDS-PAGE gel and subsequently transferred onto a polyvinylidene fluoride (PVDF) membrane (Millipore) for immunoblotting analysis using specific antibodies. The bound complexes were visualized using the NcmECL Ultra substrate (New Cell and Molecular Biotech, Suzhou, China) according to the manufacturer’s protocol.

### Adhesion assay

Yeast surface display adhesion assay was performed according to the method described by Wang et al. [25]. For the adhesion assay, DF-1 cells were incubated with 100 EtSERPIN1 displaying yeasts for 2 h at 30 °C. The cells were subsequently washed three times with PBS and plated on synthetic defined medium with casamino acids (SDCAA: 0.67% yeast extract, 2% glucose, 0.5% casein acid hydrolysate, 0.15% agar powder, and 10% ampicillin) containing 0.8% agar. After culturing for 24 h, the adhesion rate was calculated as previously described [11]. Yeasts transfected with pCTCON2 plasmid were used as the control. Adhesion inhibition assays were conducted using two methods: first, yeast cells displaying EtSERPIN1 were pre-incubated with different concentrations of recombinant His-tag GgANXA2 protein (rHis-GgANXA2) for one hour before being added to DF-1 cells for calculation of the adhesion rate; in the other, DF-1 cells were incubated with anti-ANXA2 pAb, and then yeast displayed EtSERPIN1 were added into DF-1 cells for adhesion rate calculation. Yeasts or DF-1 treated with an equivalent dose of PBS or healthy IgG served as controls. The experiment was repeated three times.

### Binding of EtSERPIN1 to chicken caecum

The ability of EtSERPIN1 to bind to chicken caecum was determined using immunohistochemical assay [26]. Briefly, the caecum was washed with sterile PBS, fixed with 4% paraformaldehyde and embedded in paraffin. Subsequently, the samples were boiled for 10 min in sodium citrate buffer (Servicebio, Wuhan, China), followed by blocking with tris-buffered saline and polysorbate 20 (TBST) containing 5% bovine serum albumin (BSA). Next, tissue sections were incubated sequentially with rHis-EtSERPIN1 or PBS (negative control) at 37 °C for 1 h. Afterwards, the sections were incubated overnight at 4 °C with anti-EtSERPIN1 pAb as a primary antibody and then treated with FITC-conjugated goat anti-mouse IgG as a secondary antibody at 37 ℃ for another hour. Nuclei were stained with DAPI (Solarbio) for 5 min. For the binding inhibition assay, the sections were pre-treated with anti-ANXN2 pAb for 1 h at 37 °C. Images were visualized and acquired using fluorescence microscopy (Nikon-ECLIPSE; Japan).

### Detection of the binding between GgANXA2 and sporozoite

Sporozoites were pre-incubated with rHis-GgANXA2 protein for one hour at 4 °C. For western blot analysis, the sporozoites were lysed on ice with a lysis buffer (50 mM Tris–Cl, pH 7.4, 150 mM NaCl, 1% Triton X-100, 1% sodium deoxycholate, 1 mM, Na3VO4, 1 mM EDTA, and 1 mM PMSF) for one hour, followed by three washes. For immunofluorescence assays, sporozoites were washed sequentially, fixed onto glass slides, and blocked with PBST containing 2% BSA. The samples were then incubated overnight at 4 °C with anti-His monoclonal antibody (mAb), followed by incubation with FITC-conjugated secondary antibodies at 37 °C for one hour. The samples were subsequently stained with DAPI (Solarbio) for 5 min. After washing with PBS, the samples were examined using fluorescence microscopy (Nikon, Japan).

### Sporozoite invasion assay

The impact of GgANXA2 on sporozoite invasion of host cells was investigated using an in vitro and in vivo sporozoite invasion inhibition assay [22]. In the in vitro assay, sporozoites were incubated with varying doses of rHis-GgANXA2 (50, 100, 150 μg/mL) or DF-1 cells were blocked by anti-GgANXA2 pAb (0, 100, 200, 300 μg/mL) respectively at 37 ℃ for 2 h. After three washes with PBS, the pretreated sporozoites (2 × 10^5^) were added to a 12-well plate containing DF-1 cells and unpretreated sporozoites were used to invade pretreated DF-1 cells. Following a 40 min incubation period, non-invaded sporozoites were harvested and counted. The inhibition rates were calculated as follows: (1-the number of unblocked sporozoites/the total sporozoites) × 100%. Sporozoites incubated with PBS or DF-1 incubated with the same dose of mouse IgG served as the negative control. The experiment was conducted three times. For the in vivo assay, 1 × 10^6^ sporozoites pretreated with rHis-GgANXA2 or PBS, were inoculated to chickens by cloacal injection. The cecal lesion score and cecal parasite load were detected at 5 days post-infection (dpi) as described previously [11, 27]. The oocyst output were counted at 7–10 dpi [28], respectively.

### Reverse transcription and quantitative real-time PCR (qPCR)

The chickens were orally infected with sporulated *E. tenella* oocysts (2 × 10^4^/chicken) for 0, 12, 24 h. Total RNA was isolated from the duodenum, jejunum, ileum, and caecum using an RNA purification Kit (Promega, Madison, WI, USA). Subsequently, reverse transcription of 1 μg RNA to cDNA was performed using M-MLV reverse transcriptase with RNase inhibitor. QPCR analysis was conducted in triplicate using RealStar Green Fast Mixture (A303; GenStar, Beijing, China) on an ABI Q5 instrument (ABI, Thermo Fisher Scientific Inc., MA, USA). Threshold cycle numbers were normalized to triplicate samples amplified with *GgGAPDH* gene. The qPCR primers used for *GgANXA2* and *GgGAPDH* gene are listed in Table 1.

**Table 1:** **Primers used in this study**.

Gene	Forward sequence (5’-3’)	Reverse sequence (5’-3’)
*GgANXA2*	AGGGCCTGGGAACTGATGAA	GCCAGGGCAACCATTAGCTT
*GgGAPDH*	CACCGCTATTCCTTATAAAGAAAGT	AAACACTTTCTTTATAAGGAATAGC

### Protective effect of rHis-EtSERPIN1 on *E. tenella* infection

Seven-day-old chickens were randomly divided into three groups (*n* = 35/group). The immune program was carried out as described previously [11]. In brief, chickens of the EtSERPIN1 group were immunized subcutaneously with 50 μg/chicken purified rHis-EtSERPIN1 in Freund complete adjuvant (FCA; Sigma). Group PBS-I and PBS-II were immunized with PBS-FCA. At 7 days post-immunization, chickens were administered a booster immunization of rHis-EtSERPIN1 in Freund incomplete adjuvant (FIA; Sigma) or PBS-FIA respectively. At seven days after secondary immunization, all chickens, except for the PBS-I group, were orally infected with *E. tenella* sporulated oocysts (1 × 10^4^ per chicken).

The protective efficacy was evaluated by calculated anticoccidial index (ACI) based on survival rate, weight gains, oocyst output and cecal lesion score. The body weight gains in each group were determined during 0 dpi to 10 dpi. Oocyst output was estimated by counting the oocysts per gram of feces during 7 dpi and 10 dpi. The chicken cecal lesion scores (*n* = 8) were recorded at 5 dpi as described previously. ACI of each group was evaluated as described previously, using the following formula ACI = (the relative body weight gain + survival rate) − (oocyst count index + lesion score index) [29].

### Serum IgG and cecal sIgA level

The levels of serum IgG and cecal secretory IgA (sIgA) in each group of chickens (*n* = 3) were determined using ELISA, according to the protocol described previously [30]. Briefly, chickens were killed by cervical dislocation, and the ceca were carefully removed and cut longitudinally. The ceca were then incubated for 4 h on ice in 10 mL of ice-cold PBS, containing 0.05 trypsin inhibitory units/mL of aprotinin, 5.0 mM EDTA, 2.0 mM phenylmethylsulfonyl fluoride, and 0.02% NaN3 for preparation of sIgA. ELISA plates coated with recombinant His-EtSERPIN1 protein were used to quantify IgG in serum and sIgA in caeca. Horseradish peroxidase-conjugated rabbit anti-chicken IgG antibodies were employed as the secondary antibodies. All samples were analyzed in triplicate, and the optical density at 450 nm was measured using an automated microplate reader (Biotek, USA).

### Data statistical analysis

The independent experiments were conducted in triplicate, with three technical replicates for each experiment. All statistical analyses were performed using GraphPad Prism 9.0 software. A student *t*-test was utilized to determine the significance of the results, and the data were presented as mean ± SD. *P* value < 0.05 was considered statistically significant.

## Results

### EtSERPIN1 expressed on the surface of sporozoites is involved in adhesion and invasion process

**Figure 1:**
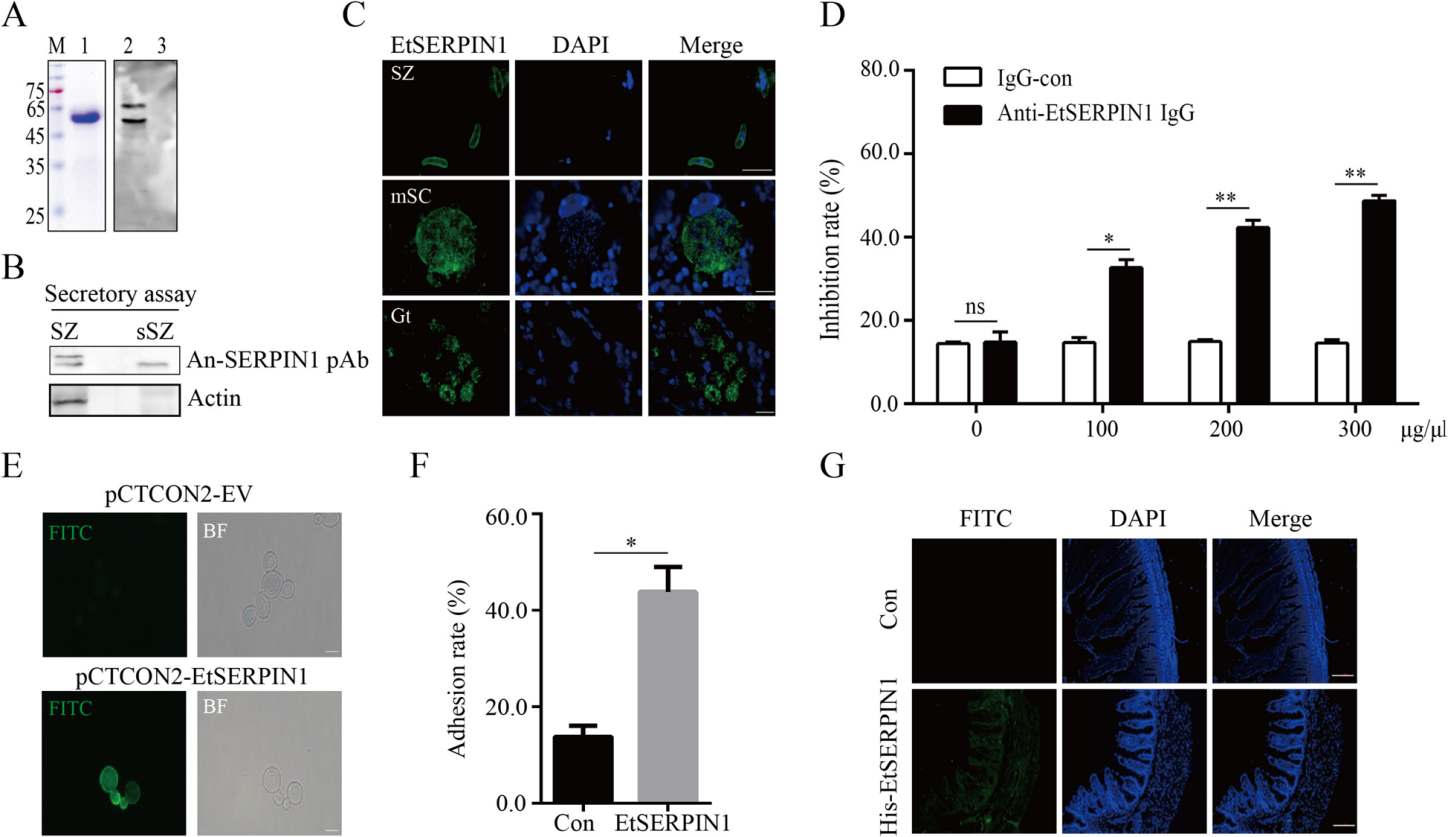
**EtSERPIN1expressed on the surface of sporozoite is involved in the adhesion process**. **A** SDS-PAGE analysis of purified recombinant His-EtSERPIN1 protein (lane 1). EtSERPIN1 expressed in sporozoites of *E. tenella* was detected by western blot using an anti-EtSERPIN1 pAb as the primary antibody (lane 2), with chicken cecum protein used as a control (lane 3). **B** Western blot analysis of whole sporozoite proteins and secreted sporozoite proteins, using anti-EtSERPIN1 pAb and anti-GAPDH pAb. GAPDH was used as a loading control. **C** Localization of EtSERPIN1 in *E. tenella* sporozoites (SZ), mature schizonts (mSC) and gametophytes (Gt) by IFA, using anti-EtSERPIN1 pAb as primary antibody. Scale bars, 50 μm. **D** Inhibition of sporozoite invasion by anti-EtSERPIN1 pAb at various concentrations. **E** Surface expression of EtSERPIN1 on yeast cells was confirmed by IFA. **F** Adhesion rate of yeast cells displaying EtSERPIN1 to host cells. Scale bars, 10 μm. **G** Cecum tissues were incubated with or without His-EtSERPIN1 protein, and His-EtSERPIN1 was detected using an anti-His mAb as the primary antibody and FITC-conjugated goat anti-mouse antibody as the secondary antibody. The nucleus was stained with DAPI. Scale bars, 50 μm. *P < 0.05, **P < 0.01.

It has been previously reported that EtSERPIN1 is expressed in both sporozoites and schizogony stages [14]. However, the expression of EtSERPIN1 during the gametogony stage and its localization in sporozoites remain unclear. To clarify the expression and localization of EtSERPIN1, we expressed and purified rHis-EtSERPIN1 with a molecular weight of approximately 50 kDa (Figure 1A, lane 1) to prepare anti-EtSERPIN1 polyclonal antibody (pAb). Anti-EtSERPIN1 pAb prepared in mice recognized two protein bands of sporozoite protein at around 42 kDa and 45 kDa (Figure 1A, lane 2). Further investigation revealed a secreted protein band at around 42 kDa in sporozoite secretion proteins (Figure 1B), suggesting that native EtSERPIN1 may exist in two forms in *E. tenella*, with the lower molecular weight form being a secreted protein lacking signal peptide and cytoplasmic domain. Using anti-EtSERPIN1 pAb, we determined the expression and localization of EtSERPIN1 during the sporozoite invasion process as well as gametogony stage. Immunofluorescence results found that EtSERPIN1 has been labeled on the surface of sporozoites and expressed in the schizonts and gametogony stages (Figure 1C). These results indicate that EtSERPIN1 is a membrane and secreted protein that is expressed in all life stages of *E. tenella*.

Previous studies have demonstrated the inhibitory effect of anti-EtSERPIN1 antibody on sporozoite invasion, suggesting its involvement in the parasite invasion process [14]. Our antibody inhibition test results also revealed that pretreatment with anti-EtSERPIN1 pAb effectively reduced the invasion ability of parasites in DF-1 cells in a dose-dependent manner (Figure 1E). At a concentration of 300 μg/mL, anti-EtSERPIN1 pAb blocked 48.77% of sporozoites from invading DF-1 cells, which was significantly higher than the control (14.6%) (*P* < 0.05) (Figure 1D). Additionally, to elucidate the role of surface EtSERPIN1, we investigated its impact on sporozoite adhesion and invasion processes. The binding ability of EtSERPIN1 was assessed using yeast display system and immunohistochemical assays as described in the Materials and methods. Yeast expressing surface-bound EtSERPIN1 (Figure 1E) exhibited significantly enhanced adhesion to DF-1 cells compared to controls (Figure 1F) (*P* < 0.05). Immunohistochemistry confirmed specific binding between rHis-EtSERPIN1 and cecum tissue sections labeled with anti-EtSERPIN1 pAb (Figure 1G), further supporting the adhesive function of EtSERPIN1.

### EtSERPIN1 interacts with GgANXA2

**Figure 2:**
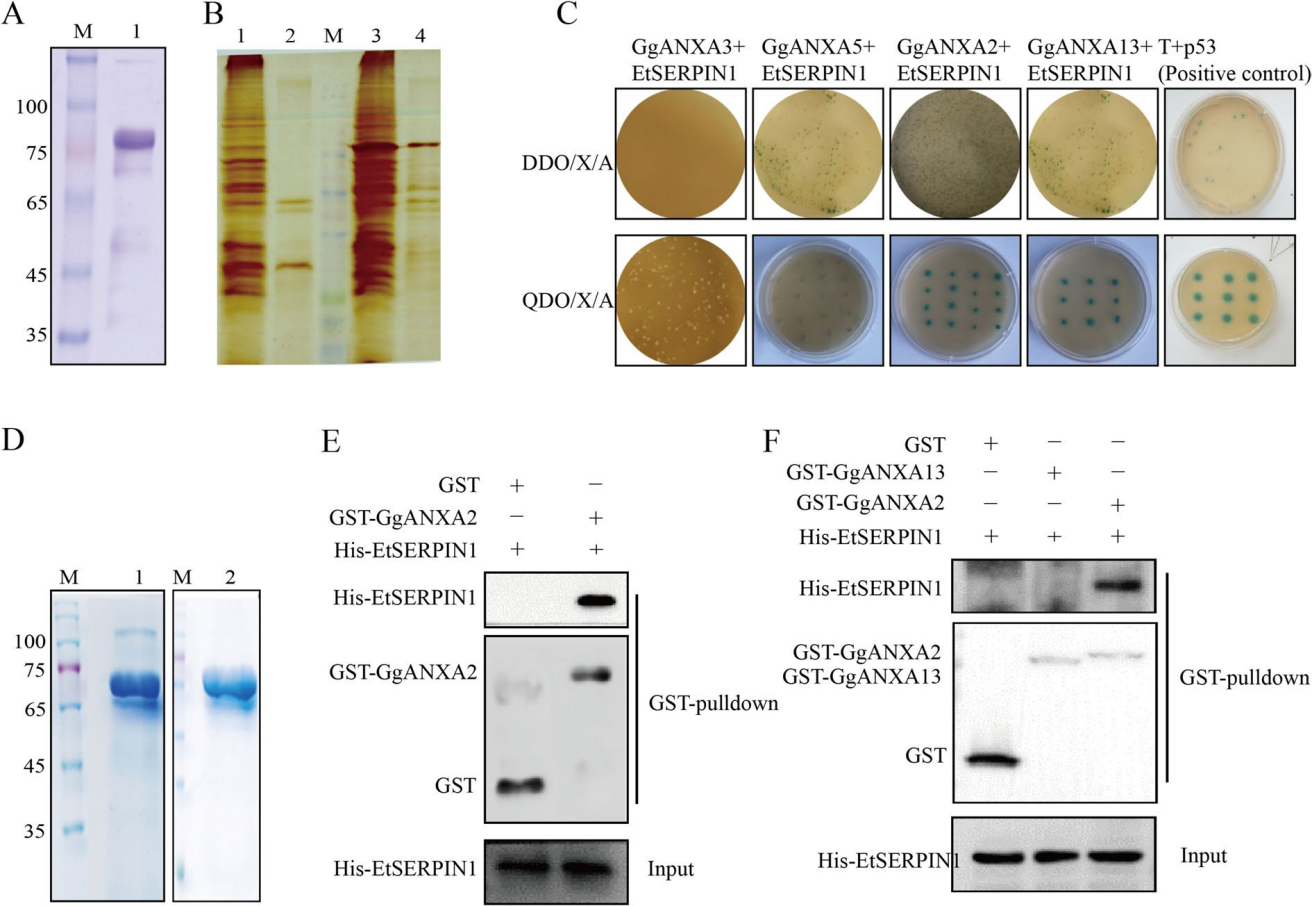
**EtSERPIN1 interacts with GgANXA2. A** SDS-PAGE analysis of purified rGST-EtSERPIN1 protein. **B** SDS-PAGE identification of cecal membrane proteins interacting with rGST-EtSERPIN1. M: protein marker; Lane 1: GST-tag protein expressed in *E. coli* BL21; Lane 2: GST-pull-down products of GST-tag; Lane 3: rGST-EtSERPIN1 expressed in *E. coli* BL21; Lane 4: GST-pull-down products of rGST-EtSERPIN1. **C** Yeast two-hybrid (GY2H) assay to test interactions between EtSERPIN1 and host proteins. p53 was used as a positive control. **D** SDS-PAGE analysis of purified rGST-GgANXA2 and rGST-GgANXA13 proteins. **E**, **F** GST pull-down assays to detect interactions between rHis-EtSERPIN1 and rGST-GgANXA2 (E) or rGST-GgANXA13 (**F**). The binding of rHis-EtSERPIN1 to GST-GgANXA2 or GST-GgANXA13 was confirmed by western blotting using anti-His monoclonal antibody (mAb) or anti-GST mAb.

To identify the binding protein of EtSERPIN1 associated with adhesion and invasion processes, the membrane protein of chicken cecal epithelial cells interacting with EtSERPIN1 were screened and identified by GST pull-down and MS analysis. The recombinant EtSERPIN1 protein tagged with GST was successfully expressed and purified (Figure 2A). Differential protein bands were observed in the rGST-EtSERPIN1 pull-down mixture compared to the GST-tag control (Figure 2B). MS analysis revealed a total of 27 candidate host proteins potentially associated with EtSERPIN1. Subsequently, we predicted the sub-cellular localization of these 27 proteins using WoLF PSORT analysis and UniProt database, which identified four proteins located on the cell membrane, including GgANXA2/3/5/13. Considering that adhesion and invasion processes are mediated by interactions between parasite proteins and surface receptors on host cells, we selected these four membrane proteins as candidates for further identification studies.

To validate the interaction between EtSERPIN1and four membrane proteins, a point-to-point Y2H assay was performed. Yeast cells transformed with pGBKT7-EtSERPIN1 and either pGADT7-GgANXA2 or pGADT7-GgANXA13 displayed blue colonies on DDO/X/A and QDO/X/A media, while those transformed with GgANXA3 or GgANXA5 constructs did not exhibit any color change (Figure 2C), indicating potential interactions between EtSERPIN1 and GgANXA2/GgANXA13. Additionally, a GST pull-down assay was conducted to confirm direct interactions between EtSERPIN1 and GgANXA2/GgANXA13. rGST-GgANXA2 and rGST-GgANXA13 protein were expressed and purified successfully (Figure 2D). The results from the GST pull-down experiment demonstrated that His-EtSERPIN1 could be pulled down by rGST-GgANXA2 but not by the negative control containing only GST tag (Figure 2E). However, no interaction between rHis-EtSERPIN1 was observed in the eluate obtained from the rGST-GgANX13 pull-down experiment (Figure 2F). These findings provide evidence for an interaction between EtSERPIN1 and GgANXA2, a chicken membrane protein.

### Recombinant GgANXA2 protein binds to sporozoites

**Figure 3:**
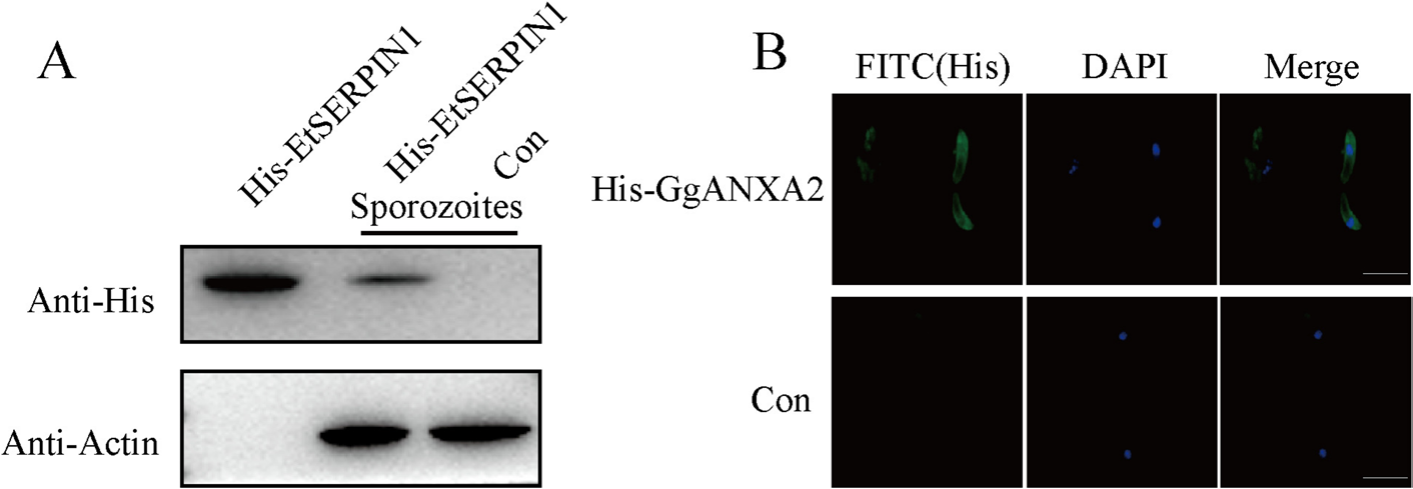
**Recombinant GgANXA2 protein bind to the surface of sporozoites.** *E. tenella* sporozoites were incubated with rHis-GgANXA2 protein and subsequently analyzed by western blotting (**A**) and immunofluorescence assay (IFA) (**B**), using an anti-His mAb as the primary antibody. Scale bars, 50 μm.

To further validate the interaction between EtSERPIN1 and GgANXA2, we initially incubated sporozoites with rHis-GgANXA2 protein and employed western blot to assess the binding affinity of rHis-GgANXA2 protein towards sporozoites. The western blot analysis revealed detectable levels of rHis-GgANXA2 in sporozoites incubated with rHis-GgANXA2, while no signal was observed in the control group (Figure 3A). Additionally, IFA using anti-His mAb demonstrated surface localization of sporozoites incubated with rHis-GgANXA2 protein (Figure 3B). These findings provide compelling evidence that GgANXA2 effectively interacts with the surface of sporozoites, thereby confirming its association with EtSERPIN1.

### Recombinant GgANXA2 protein and GgANXA2-specific antibody inhibited EtSERPIN1 binding to host cells

**Figure 4:**
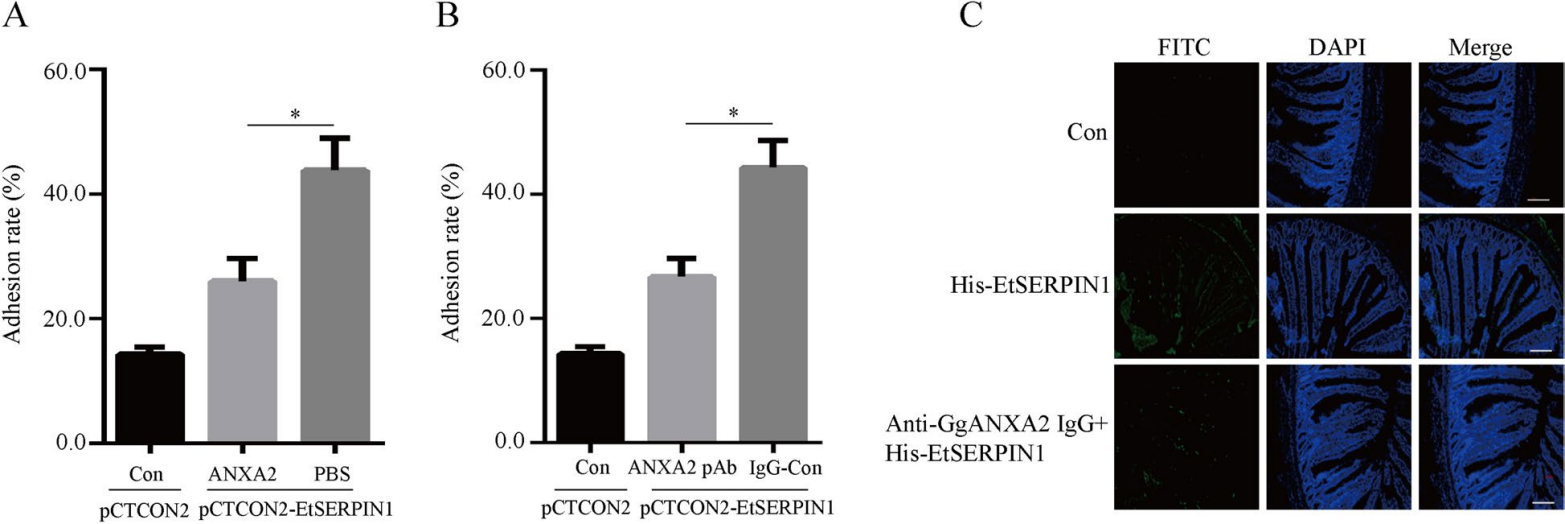
**Recombinant GgANXA2 protein and anti-GgANXA2 specific antibody inhibit EtSERPIN1 binding to the host cell. A** Yeast cells displaying EtSERPIN1 were pre-incubated with recombinant GgANXA2 protein or PBS (control). The adhesion rate of yeast cells was then measured. **B** DF-1 cells were pre-treated with anti-ANXA2 pAb or IgG (control). Yeast cells displaying EtSERPIN1 or an empty plasmid were subsequently added to the cells, and the adhesion rate of yeast cells was calculated. *P < 0.05. **C** Cecal tissues were pre-treated with anti-ANXA2 pAb. The samples were incubated with or without rHis-EtSERPIN1 protein, followed by detection of rHis-EtSERPIN1 using anti-His mAb as the primary antibody and FITC-conjugated goat anti-mouse antibody as the secondary antibody. Nuclei were stained with DAPI. Scale bars, 50 μm.

To further elucidate the role of GgANXA2 in EtSERPIN1-mediated adhesion to host cells, we conducted an attachment assay using the yeast surface display model. The adhesion rate of yeast cells displaying EtSERPIN1 was significantly reduced in the presence of recombinant GgANAX2 proteins (44.32%) compared to the PBS group (26.75%) (*P* < 0.05) (Figure 4A). We performed cell blocking with anti-ANAX2 antibody and assessed its impact on EtSERPIN1 adhesion. Pre-blocking cells with anti-ANXA2 antibody resulted in a significantly lower adhesion rate compared to incubation with IgG alone (Figure 4B). Immunohistochemistry assays revealed that rHis-EtSERPIN1 was capable of binding to cecum tissue (Figure 4C). However, when pre-incubated with anti-GgANXA2 IgG, the binding of rHis-EtSERPIN1 to the cecum tissue was significantly reduced (Figure 4C), indicating that anti-GgANXA2 IgG effectively blocked rHis-EtSERPIN1 binding to the tissue. These results suggest that GgANXA2 may be essential for mediating EtSERPIN1’s adherence to host cells.

### Recombinant GgANXA2 protein inhibited sporozoite invasion in vitro and in vivo

**Figure 5:**
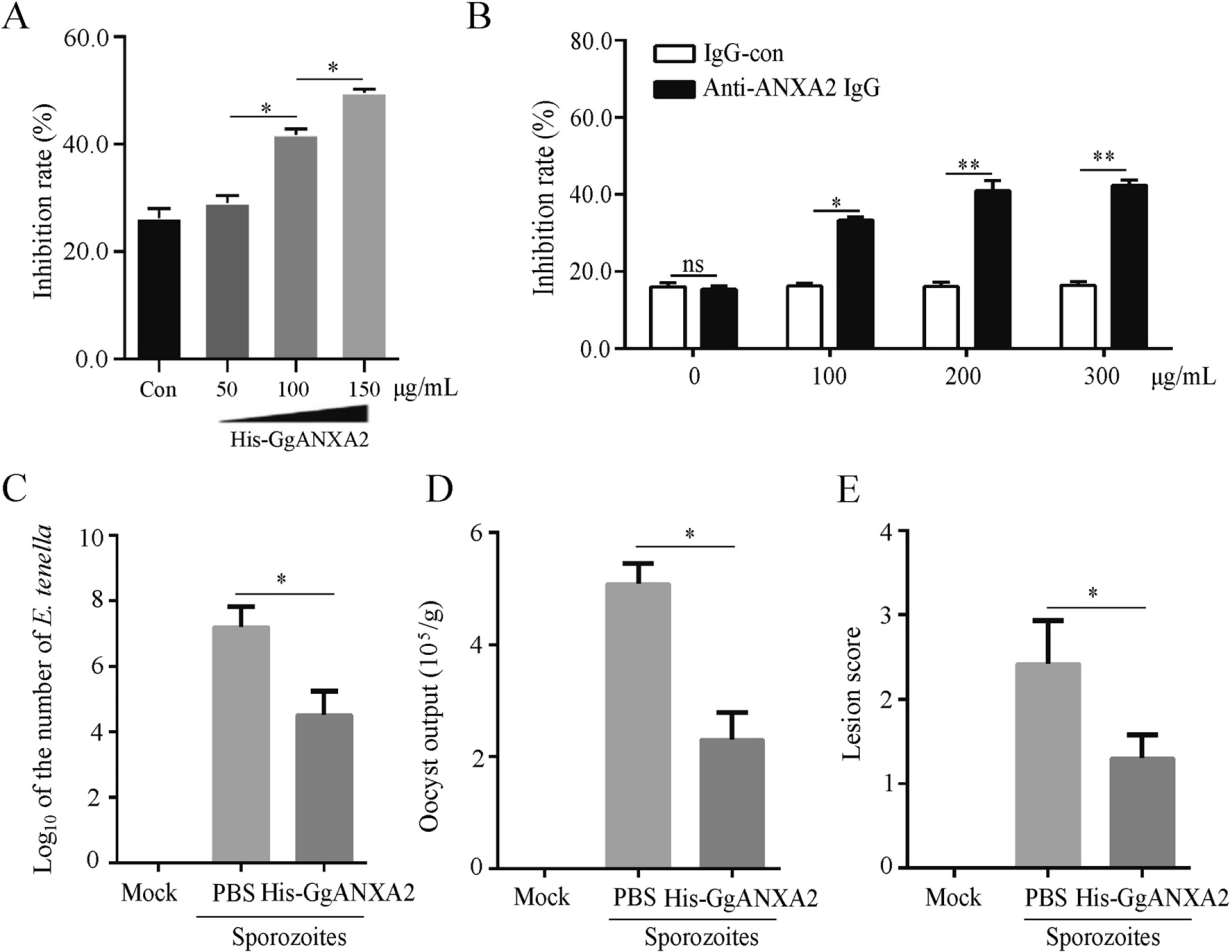
**Recombinant GgANXA2 protein and anti-GgANXA2 specific antibody inhibits sporozoite invasion in vitro and in vivo. A** Sporozoites of *E. tenella* were incubated with rHis-GgANXA2 protein at different concentrations. The invasion rate of sporozoites was then measured. **B** DF-1 cells were treated with anti-ANXA2 pAb at various concentrations, followed by infection with sporozoites, and the invasion rate was calculated. **C**-**E** *E. tenella* sporozoites were incubated with rHis-GgANXA2 protein, and then infected via the rectum. At 5 days post-infection (dpi), parasite load in the cecum was determined by qPCR (**C**), and lesion scores were recorded (**D**). The number of oocysts per gram of feces was counted from 7 to 10 dpi. *P < 0.05, **P < 0.01.

Furthermore, we investigated the impact of GgANXA2 recombinant protein and anti-ANAX2 antibody on sporozoite invasion both in vitro and in vivo. The findings demonstrate that incubation with recombinant GgANXA2 protein or cell blocking using anti-ANXA2 antibodies significantly reduced the invasion rate of sporozoites in a dose-dependent manner (Figures 5A and B). These results indicate that both GgANXA2 protein and anti-ANXN2 antibody blockade effectively inhibit the invasion of sporozoites into host cells.

In vivo experiments evaluated the effect of GgANXN2 recombinant protein on sporozoite infection, including cecal load, cecal lesions, and fecal oocyst excretion. Compared to the control group, there was a significant reduction in *E. tenella* parasite load within the cecum (Figure 5C) as well as oocyst excretion following sporozoites incubated with recombinant GgANXA2 protein (Figure 5D). In vivo, Additionally, at 5 days post-infection (dpi), cecal lesion scores were significantly lower in the GgANXA2 protein incubation group compared to those observed in the control group (Figure 5E). These findings suggest that GgANXA2 protein blockade possesses inhibitory potential against sporozoite infection.

### The expression of GgANXA2 in the cecum is regulated by* E. tenella* infection

**Figure 6:**
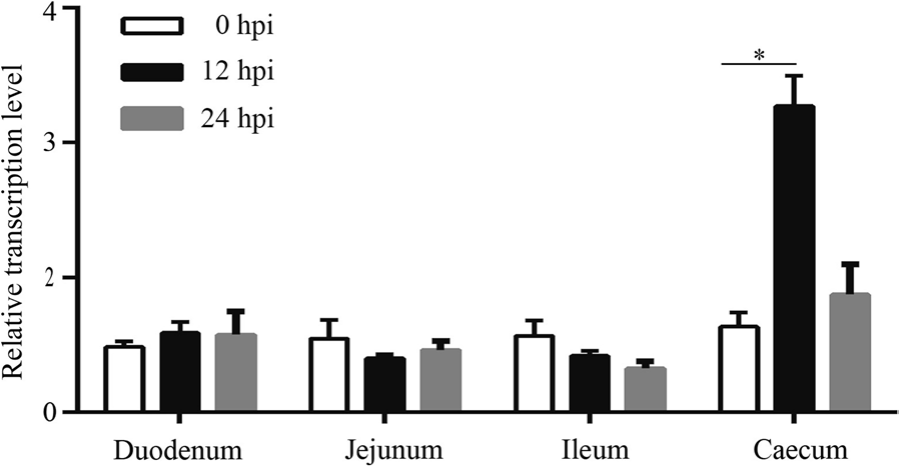
**Upregulation of GgANXA2 transcription following *E. tenella* infection.** The mRNA levels of GgANXA2 in the duodenum, jejunum, ileum, and caecum of chickens were quantified by qPCR at 0, 12, and 24 h post-infection (hpi). *P < 0.05 indicates statistically significant differences.

Further, we examined the transcriptional levels of GgANXA2 in the duodenum, jejunum, ileum and caecum during *E. tenella* infection. The results of qPCR revealed no significant difference in GgANXA2 transcriptional levels between the duodenum, jejunum, and ileum at 12 h post-infection (hpi). However, there was a significant increase in GgANXA2 expression in the cecum compared to 0 hpi (Figure 6). These findings suggest a positive association between cecal ANXA2 transcription and sporozoite invasion.

### Recombinant EtSERPIN1 immunization induced higher anti-*E. tenella* activity

Considering the involvement of EtSERPIN1 in the adhesion and invasion process of *E. tenella*, we postulated that antibodies induced by EtSERPIN1 possess anti-*E. tenella* activity. Subsequently, we evaluated the anti-*E. tenella* activity elicited by immunization with rHis-EtSERPIN1 protein through assessments of survival rate, body weight, cecal lesions, and oocyst count. Throughout the experiment, no deaths occurred due to *E. tenella* infection in any group, resulting in a 100% survival rate. Remarkably increased relative weight gain was observed in chickens immunized with rHis-EtSERPIN1 protein compared to those in the control group (Table 2). Additionally, chickens receiving rHis-EtSERPIN1 protein exhibited significantly lower cecal lesion scores than those in the control group (*P* < 0.05). Moreover, oocyst shedding was significantly reduced in chickens immunized with EtSERPIN1 compared to PBS controls. Finally, ACI calculation results revealed an anti-*E. tenella* index of 170.14 for rHis-EtSERPIN1 protein (Table 1). Collectively, these findings demonstrate that immunization with recombinant EtSERPIN1 protein induces a moderate level of anti-*E.* *tenella* activity in chickens and supports its candidacy as an antigen.

**Table 2:** **Protective eN1 against *E. tenella*** **infection in chickens.**

Group	Survival rate %	Average body weight gain (g)	Relative body weight gain %	Oocyst shedding (× 10^5^/g)	Oocyst count index	Lesion score	lesion score index	ACI
PBS-I	100	167.4 ± 4.85^a^	100	0	0	0	0	200
PBS-II	100	95.4 ± 4.33^c^	56.99	6.83 ± 0.29^b^	40	3.5 ± 0.16^a^	35	81.99
EtSERPIN1	100	157.6 ± 4.95^b^	94.14	3.03 ± 0.24^a^	10	1.4 ± 0.1^b^	14	170.14

Values with different letters in the same column are significantly different (*P* < 0.05). ACI = (the relative body weight gain + the survival rate) − (lesion score index + oocyst count index). Excellent activity: ACI > 180; moderate activity: 179 > ACI > 160; limited activity: 159 > ACI > 120; nonactivity: ACI < 120.

### Recombinant EtSERPIN1 induced high levels of IgG and sIgA

**Figure 7:**
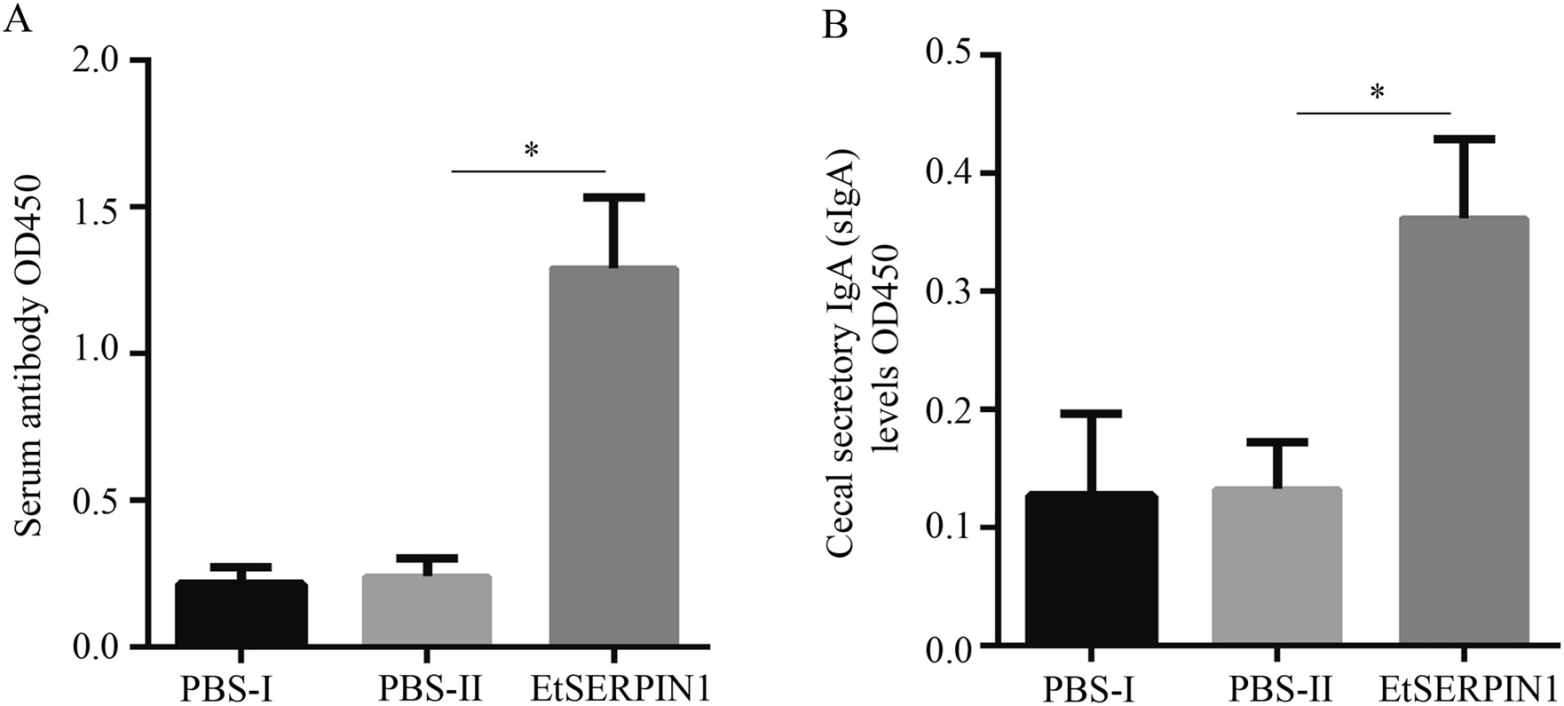
**Serum IgG and cecal sIgA levels. A** The serum IgG antibody levels against EtSERPIN1 were measured seven days after the second immunization. **B** The cecal sIgA antibody levels against EtSERPIN1 were measured at 10 dpi. *P < 0.05 denotes statistically significant differences.

To assess the capacity of EtSERPIN1 to elicit humoral immunity, we measured the serum IgG and cecal secretory IgA (sIgA) levels specific to EtSERPIN1 seven days after the second immunization. As depicted in Figure 7, the EtSERPIN1-immunized group exhibited significantly elevated levels of IgG in serum (Figure 7A) and sIgA (Figure 7B) in the cecum. These findings indicate that EtSERPIN1 possesses the ability to stimulate both humoral and mucosal immune responses.

## Discussion

Chick *Eimeria* spp. encompasses various developmental stages, with gene expression in each stage typically linked to their respective functions. Previous studies have reported the expression of EtSEPRIN1 in oocysts, sporozoites, and merozoites [14]; however, its expression during the gamete stage remains unknown. In this study, indirect immunofluorescence assay revealed that EtSERPIN1 is also expressed during gamete development, indicating its continuous expression throughout the developmental history of *E. tenella* and suggesting potential multifunctionality for EtSERPIN1. Bioinformatics analysis further identified a signal peptide and transmembrane domain in EtSERPIN1 protein sequence, implying its classification as a membrane protein [14]. Additionally, our findings demonstrate the localization of EtSERPIN1 on the sporozoite membrane which aligns with theoretical predictions and provides insights into its role in adhesion invasion.

The coding sequence of EtSERPIN1 produces a protein of approximately 45 kDa [14]. However, the anti-EtSERPIN1 antibody detected two protein bands with different molecular weights in sporozoite whole proteins, consistent with previous findings on *T. gondii* SERPIN [19]. Previous evidence suggests that *T. gondii* secretes SERPIN into host cells to prevent protease degradation [19]. Based on this, we hypothesize that immature EtSERPIN1 exists in the parasite cytoplasm as a large band without protease inhibitory activity, while mature and cleaved EtSERPIN1 is secreted extracellularly to perform its function, resulting in smaller molecular weight. Fetterer et al. reported the detection of EtSERPIN1 in host cells after sporozoite infection, and our study shows expression of surface-bound and secretory properties for EtSERPIN1 on *E. tenella* sporozoites [15], suggesting a potential for protease inhibition by externally-secreted or cell-surface bound EtSERPIN1.

The invasion process of *E. tenella*, a highly pathogenic parasite that causes significant economic losses to the poultry industry, is complex and involves multiple proteins [31, 32]. The role of secretory proteins, particularly in the early stages of invasion, has garnered considerable attention due to their potential to facilitate parasite entry into host cells. Previous studies have reported crucial roles played by rhoptry proteins and microneme proteins in the invasion process of apicomplexan protozoan parasites [32, 33]. Among these, microneme proteins (MIC) are primarily involved in the adhesion process of sporozoites to host cells, such as MIC2, MIC3 and MIC8, etc. [11, 12, 34]. The rod-neck protein forms a moving junction with apical membrane antigen (AMA1), mediating the invasion process of the parasite including rhoptry neck protein 2, 4,5, and 8 (RON2/4/5/8) [35, 36]. However, our understanding of sporozoite invasion processes in *Eimeria* spp remains extremely limited. A previous study demonstrated higher expression levels of EtSERPIN1 in sporozoites compared to other developmental stages [37]. Subsequent studies found that EtSERPIN1 was expressed at the apical end during sporozoite invasion [15], and anti-EtSERPIN1 antibodies were shown to block the sporozoite invasion process [14]. In our study, we also observed that anti-EtSERPIN1 antibodies reduced the rate of sporozoite invasion in a dose-dependent manner. Furthermore, we investigated the role of EtSERPIN1 for sporozoite invasion using immunohistochemistry and a yeast surface display system [22]. Our results reveal that EtSERPIN1 significantly enhanced yeast adhesion to host cells and recombinant EtSERPIN1 protein exhibited adhesive properties towards cecum tissue. The findings of this study suggest that EtSERPIN1 may play a crucial role in the invasion of sporozoites by facilitating their adhesion to host cells.

Protein–protein interactions are indispensable for the proper functioning of proteins [38]. Investigating the precise functions of invasion-related molecules in *E. tenella* and identifying their interacting partners would be a significant breakthrough in understanding the functional mechanisms of parasites and discovering novel antigen targets [39]. Recently, several studies have been conducted on invasion related molecules of *Eimeria* spp, such as EtMIC8, EtCab, and EtCDPK4, play a crucial role in the process of sporozoite invasion into the host cell [11, 25, 40]. However, the investigation of adhesion and invasion receptors associated with host epithelial cells remains limited. Zhang et al. [41] reported that *E. acervulina* microneme 3 protein (EaMIC3) specifically binds to chicken ubiquitin-binding enzyme E2F (UBE2F), thereby mediating the invasion process. Sun et al. [22] discovered that the interaction between EtMIC8-EGF and chicken epithelial cell adhesion molecule (GgEPCAM) is essential for attachment and invasion of *E. tenella* sporozoites [22]. Our study, in conjunction with previous reports, has confirmed the involvement of EtSERPIN1 in the sporozoite invasion process [14]. To elucidate the specific mechanism by which EtSERPIN1 mediates adhesion, we identified membrane proteins that interact with EtSERPIN1 through mass spectrometry, yeast two-hybrid assays, and pull-down experiments. These analyses confirmed an interaction between EtSERPIN1 and GgANXN2. Subsequent experiments revealed that disrupting the interaction between EtSERPIN1 and GgANXA2 on the host cell surface, either through recombinant GgANXA2 protein or anti-GgANXA2 antibody, significantly hinder the sporozoite invasion process. This suggests that the EtSERPIN1-GgANXA2 interaction is crucial for the sporozoite invasion process. Based on these results, EtSERPIN1 emerges as a promising drug target for the treatment of coccidiosis. However, further investigation into the specific domains of EtSERPIN1 responsible for binding to GgANXA2 is required. Such studies will provide a robust scientific foundation for the design of targeted therapeutics against EtSERPIN1.

Adhesion and invasion are crucial steps in establishing infection by apicomplexan protozoa. Therefore, host-produced antibodies against these processes can block sporozoite invasion and reduce infection rates. For instance, apical membrane antigen (AMA1) and rhoptry neck protein 4 (RON4) of *Neospora Canis* induce high titers of antibodies against neosporosis, while *Eimeria* spp’s MIC2, AMA1, MIC3, and MIC8 proteins elicit moderate to excellent anti-coccidia activity [11, 42–44]. In this study, recombinant EtSERPIN1 protein demonstrate high immunogenicity and protective efficacy as evidenced by increased body weight gains along with significantly lower fecal oocyst shedding levels and lesion scores. Consistent with these results were the increases in serum IgG levels and cecal mucosa sIgA antibody levels following immunization with recombinant EtSERPIN1 protein. Mucosal immunity is the first line of defense against infections; sIgA plays a critical role in defending against intestinal parasites challenge [45, 46]. Davis and Porter [47] found that anti-*E. tenella* sIgA present in cecal contents could disrupt parasite development highlighting the importance of mucosal immunity to *E. tenella* infection in chickens. Thus, high titers of anti-EtSERPIN1-specific sIgA antibodies contribute to resistance to *E. tenella* infection.

In conclusion, our study reveals that EtSERPIN1, a protease inhibitor in *E. tenella*, interacts with host cell membrane protein GgANXA2, playing a crucial role in sporozoite adhesion and invasion. Recombinant GgANXA2 inhibits EtSERPIN1 binding and sporozoite invasion, while EtSERPIN1 immunization exhibits anti-*E. tenella* activity. These findings suggest that targeting the EtSERPIN1-GgANXA2 interaction may offer a novel therapeutic approach for *E. tenella* treatment. Further studies are needed to elucidate the underlying mechanisms and explore potential applications.

## Data Availability

All data from this study are available from the corresponding author upon reasonable request.

## References

[1] Liu Q, Liu X, Zhao X, Zhu XQ, Suo X (2023) Live attenuated anticoccidial vaccines for chickens. Trends Parasitol 39:1087–1099 37770352 10.1016/j.pt.2023.09.002

[2] Clark EL, Tomley FM, Blake DP (2017) Are *Eimeria* genetically diverse, and does it matter? Trends Parasitol 33:231–241 27593338 10.1016/j.pt.2016.08.007

[3] Clarke L, Fodey TL, Crooks SR, Moloney M, O’Mahony J, Delahaut P, O’Kennedy R, Danaher M (2014) A review of coccidiostats and the analysis of their residues in meat and other food. Meat Sci 97:358–374 24534603 10.1016/j.meatsci.2014.01.004

[4] Witcombe DM, Smith NC (2014) Strategies for anti-coccidial prophylaxis. Parasitology 141:1379–1389 24534138 10.1017/S0031182014000195

[5] Aikawa M, Miller LH, Johnson J, Rabbege J (1978) Erythrocyte entry by malarial parasites. A moving junction between erythrocyte and parasite. J Cell Biol 77:72–82 96121 10.1083/jcb.77.1.72 PMC2110026

[6] Venugopal K, Marion S (2018) Secretory organelle trafficking in *Toxoplasma gondii*: a long story for a short travel. Int J Med Microbiol 308:751–760 30055977 10.1016/j.ijmm.2018.07.007

[7] Arredondo SA, Schepis A, Reynolds L, Kappe SHI (2021) Secretory organelle function in the plasmodium sporozoite. Trends Parasitol 37:651–663 33589364 10.1016/j.pt.2021.01.008

[8] Blumenschein TM, Friedrich N, Childs RA, Saouros S, Carpenter EP, Campanero-Rhodes MA, Simpson P, Chai W, Koutroukides T, Blackman MJ, Feizi T, Soldati-Favre D, Matthews S (2007) Atomic resolution insight into host cell recognition by *Toxoplasma gondii*. EMBO J 26:2808–2820 17491595 10.1038/sj.emboj.7601704 PMC1888667

[9] Brecht S, Carruthers VB, Ferguson DJ, Giddings OK, Wang G, Jakle U, Harper JM, Sibley LD, Soldati D (2001) The toxoplasma micronemal protein MIC4 is an adhesin composed of six conserved apple domains. J Biol Chem 276:4119–4127 11053441 10.1074/jbc.M008294200

[10] Soldati D, Dubremetz JF, Lebrun M (2001) Microneme proteins: structural and functional requirements to promote adhesion and invasion by the apicomplexan parasite *Toxoplasma gondii*. Int J Parasitol 31:1293–1302 11566297 10.1016/s0020-7519(01)00257-0

[11] Zhao N, Ming S, Sun L, Wang B, Li H, Zhang X, Zhao X (2021) Identification and characterization of *Eimeria tenella* microneme protein (EtMIC8). Microbiol Spectr 9:e0022821 34479414 10.1128/spectrum.00228-21 PMC8562341

[12] Huynh MH, Carruthers VB (2006) Toxoplasma MIC2 is a major determinant of invasion and virulence. PLoS Pathog 2:e84 16933991 10.1371/journal.ppat.0020084 PMC1550269

[13] Carruthers VB, Tomley FM (2008) Microneme proteins in apicomplexans. Subcell Biochem 47:33–45 18512339 10.1007/978-0-387-78267-6_2 PMC2847500

[14] Jiang L, Lin J, Han H, Zhao Q, Dong H, Zhu S, Huang B (2012) Identification and partial characterization of a serine protease inhibitor (serpin) of *Eimeria tenella*. Parasitol Res 110:865–874 21842392 10.1007/s00436-011-2568-0

[15] Fetterer RH, Miska KB, Jenkins MC, Barfield RC, Lillehoj H (2008) Identification and characterization of a serpin from *Eimeria acervulina*. J Parasitol 94:1269–1274 18576851 10.1645/GE-1559.1

[16] Laskowski M Jr, Kato I (1980) Protein inhibitors of proteinases. Annu Rev Biochem 49:593–626 6996568 10.1146/annurev.bi.49.070180.003113

[17] van Gent D, Sharp P, Morgan K, Kalsheker N (2003) Serpins: structure, function and molecular evolution. Int J Biochem Cell Biol 35:1536–1547 12824063 10.1016/s1357-2725(03)00134-1

[18] Bruno S, Duschak VG, Ledesma B, Ferella M, Andersson B, Guarnera EA, Angel SO (2004) Identification and characterization of serine proteinase inhibitors from *Neospora caninum*. Mol Biochem Parasitol 136:101–107 15138071 10.1016/j.molbiopara.2004.03.005

[19] Pszenny V, Angel SO, Duschak VG, Paulino M, Ledesma B, Yabo MI, Guarnera E, Ruiz AM, Bontempi EJ (2000) Molecular cloning, sequencing and expression of a serine proteinase inhibitor gene from *Toxoplasma gondii*. Mol Biochem Parasitol 107:241–249 10779600 10.1016/s0166-6851(00)00202-4

[20] Riahi Y, Siman-Tov R, Ankri S (2004) Molecular cloning, expression and characterization of a serine proteinase inhibitor gene from Entamoeba histolytica. Mol Biochem Parasitol 133:153–162 14698428 10.1016/j.molbiopara.2003.10.003

[21] Liu Q, Chen Z, Shi W, Sun H, Zhang J, Li H, Xiao Y, Wang F, Zhao X (2014) Preparation and initial application of monoclonal antibodies that recognize *Eimeria tenella* microneme proteins 1 and 2. Parasitol Res 113:4151–4161 25164275 10.1007/s00436-014-4087-2

[22] Sun L, Li C, Zhao N, Wang B, Li H, Wang H, Zhang X, Zhao X (2024) Host protein EPCAM interacting with EtMIC8-EGF is essential for attachment and invasion of *Eimeria tenella* in chickens. Microb Pathog 188:106549 38281605 10.1016/j.micpath.2024.106549

[23] Li J, Xiao Q, Tan Q, Chen J, Sun L, Chen X, Chu Z, Wu H, Zhang Z, Li H, Zhao X, Zhang X (2023) TgMORN2, a MORN family protein involved in the regulation of endoplasmic reticulum stress in *Toxoplasma gondii*. Int J Mol Sci 24:10228 37373373 10.3390/ijms241210228 PMC10299398

[24] Zhao N, Wang F, Kong Z, Shang Y (2022) Pseudorabies virus tegument protein UL13 suppresses RLR-mediated antiviral innate immunity through regulating receptor transcription. Viruses 14:1465 35891444 10.3390/v14071465 PMC9317333

[25] Wang Y, Zhou X, Wang H, Sun L, Wang B, Jiang Y, Li H, Zhang X, Li H, Zhao X (2021) The role of *Eimeria tenella* EtCab protein in the attachment and invasion of host cells. Vet Parasitol 292:109415 33780830 10.1016/j.vetpar.2021.109415

[26] Li W, Wang M, Chen Y, Chen C, Liu X, Sun X, Jing C, Xu L, Yan R, Li X, Song X (2020) EtMIC3 and its receptors BAG1 and ENDOUL are essential for site-specific invasion of *Eimeria tenella* in chickens. Vet Res 51:90 32678057 10.1186/s13567-020-00809-6 PMC7367391

[27] Johnson J, Reid WM (1970) Anticoccidial drugs: lesion scoring techniques in battery and floor-pen experiments with chickens. Exp Parasitol 28:30–36 5459870 10.1016/0014-4894(70)90063-9

[28] Song X, Li Y, Chen S, Jia R, Huang Y, Zou Y, Li L, Zhao X, Yin Z (2020) Anticoccidial effect of herbal powder “Shi Ying Zi” in chickens infected with *Eimeria tenella*. Animals (Basel) 10:1484 32846893 10.3390/ani10091484 PMC7552158

[29] Qaid MM, Al-Mufarrej SI, Azzam MM, Al-Garadi MA (2021) Anticoccidial effectivity of a traditional medicinal plant, Cinnamomum verum, in broiler chickens infected with *Eimeria tenella*. Poult Sci 100:100902 33518353 10.1016/j.psj.2020.11.071 PMC7936149

[30] Chen Z, Wang X, Zhao N, Han L, Wang F, Li H, Cui Y, Zhao X (2018) Improving the immunogenicity and protective efficacy of the EtMIC2 protein against *Eimeria tenella* infection through random mutagenesis. Vaccine 36:2435–2441 29588119 10.1016/j.vaccine.2018.03.046

[31] Zhang Y, Lai BS, Juhas M, Zhang Y (2019) *Toxoplasma gondii* secretory proteins and their role in invasion and pathogenesis. Microbiol Res 227:126293 31421715 10.1016/j.micres.2019.06.003

[32] Sparvoli D, Delabre J, Penarete-Vargas DM, Kumar Mageswaran S, Tsypin LM, Heckendorn J, Theveny L, Maynadier M, Mendonça Cova M, Berry-Sterkers L, Guérin A, Dubremetz JF, Urbach S, Striepen B, Turkewitz AP, Chang YW, Lebrun M (2022) An apical membrane complex for triggering rhoptry exocytosis and invasion in Toxoplasma. EMBO J 41:e111158 36245278 10.15252/embj.2022111158 PMC9670195

[33] Bonhomme A, Bouchot A, Pezzella N, Gomez J, Le Moal H, Pinon JM (1999) Signaling during the invasion of host cells by *Toxoplasma gondii*. FEMS Microbiol Rev 23:551–561 10525166 10.1111/j.1574-6976.1999.tb00413.x

[34] Zhang D, Jiang N, Chen Q (2019) ROP9, MIC3, and SAG2 are heparin-binding proteins in *Toxoplasma gondii* and involved in host cell attachment and invasion. Acta Trop 192:22–29 30664845 10.1016/j.actatropica.2019.01.001

[35] Takemae H, Kobayashi K, Sugi T, Han Y, Gong H, Ishiwa A, Recuenco FC, Murakoshi F, Takano R, Murata Y, Nagamune K, Horimoto T, Akashi H, Kato K (2018) *Toxoplasma gondii* RON4 binds to heparan sulfate on the host cell surface. Parasitol Int 67:123–130 29081389 10.1016/j.parint.2017.10.008

[36] Straub KW, Peng ED, Hajagos BE, Tyler JS, Bradley PJ (2011) The moving junction protein RON8 facilitates firm attachment and host cell invasion in *Toxoplasma gondii*. PLoS Pathog 7:e1002007 21423671 10.1371/journal.ppat.1002007 PMC3053350

[37] Ng ST, Sanusi Jangi M, Shirley MW, Tomley FM, Wan KL (2002) Comparative EST analyses provide insights into gene expression in two asexual developmental stages of *Eimeria tenella*. Exp Parasitol 101:168–173 12427472 10.1016/s0014-4894(02)00109-1

[38] Pratt EP, Owens JL, Hockerman GH, Hu CD (2016) Bimolecular fluorescence complementation (BiFC) analysis of protein-protein interactions and assessment of subcellular localization in live cells. Methods Mol Biol 1474:153–170 27515079 10.1007/978-1-4939-6352-2_9

[39] Blake DP, Tomley FM (2014) Securing poultry production from the ever-present *Eimeria* challenge. Trends Parasitol 30:12–19 24238797 10.1016/j.pt.2013.10.003

[40] Lv L, Huang B, Zhao Q, Zhao Z, Dong H, Zhu S, Chen T, Yan M, Han H (2018) Identification of an interaction between calcium-dependent protein kinase 4 (EtCDPK4) and serine protease inhibitor (EtSerpin) in *Eimeria**tenella*. Parasit Vectors 11:259 29688868 10.1186/s13071-018-2848-y PMC5913893

[41] Zhang Z, Zhou Z, Huang J, Sun X, Haseeb M, Ahmed S, Shah MAA, Yan R, Song X, Xu L, Li X (2020) Molecular characterization of a potential receptor of *Eimeria**acervulina* microneme protein 3 from chicken duodenal epithelial cells. Parasite 27:18 32195662 10.1051/parasite/2020014 PMC7083106

[42] Zhang J, Chen P, Sun H, Liu Q, Wang L, Wang T, Shi W, Li H, Xiao Y, Wang P, Wang F, Zhao X (2014) Pichia pastoris expressed EtMic2 protein as a potential vaccine against chicken coccidiosis. Vet Parasitol 205:62–69 25047705 10.1016/j.vetpar.2014.06.029

[43] Li J, Wang F, Ma C, Huang Y, Wang D, Ma D (2018) Recombinant lactococcus lactis expressing *Eimeria tenella* AMA1 protein and its immunological effects against homologous challenge. Exp Parasitol 191:1–8 29890444 10.1016/j.exppara.2018.05.003

[44] Zhao N, Lv J, Lu Y, Jiang Y, Li H, Liu Y, Zhang X, Zhao X (2020) Prolonging and enhancing the protective efficacy of the EtMIC3-C-MAR against *Eimeria**tenella* through delivered by attenuated salmonella typhimurium. Vet Parasitol 279:109061 32143014 10.1016/j.vetpar.2020.109061

[45] Konjufca V, Jenkins M, Wang S, Juarez-Rodriguez MD, Curtiss R 3rd (2008) Immunogenicity of recombinant attenuated *Salmonella enterica* serovar Typhimurium vaccine strains carrying a gene that encodes *Eimeria tenella* antigen SO7. Infect Immun 76:5745–5753 18809658 10.1128/IAI.00897-08 PMC2583560

[46] Carrero JC, Cervantes-Rebolledo C, Aguilar-Díaz H, Díaz-Gallardo MY, Laclette JP, Morales-Montor J (2007) The role of the secretory immune response in the infection by *Entamoeba histolytica*. Parasite Immunol 29:331–338 17576362 10.1111/j.1365-3024.2007.00955.x

[47] Davis PJ, Porter P (1979) A mechanism for secretory IgA-mediated inhibition of the cell penetration and intracellular development of *Eimeria tenella*. Immunology 36:471–477 437838 PMC1457584

